# The Visual Acuity of Rats in Touchscreen Setups

**DOI:** 10.3390/vision4010004

**Published:** 2019-12-31

**Authors:** Els Crijns, Hans Op de Beeck

**Affiliations:** 1 Laboratory for Biological Psychology, Department of Brain and Cognition, KU Leuven, Tiensestraat 102 box 3714, 3000 Leuven, Belgium; elscrijns@hotmail.com; 2 Leuven Brain Institute, 3000 Leuven, Belgium

**Keywords:** visual acuity, rodent, pairwise discrimination, touchscreens

## Abstract

Touchscreen setups are increasingly used in rodents for a wide range of cognitive tasks, including visual discrimination. The greater automation and high throughput of this platform could greatly facilitate future vision research. However, little information is available regarding decision distance and on the limitations of stimulus size. Especially when studying visual functions, the lack of control of basic visual properties is a drawback. Therefore, we determined the maximal number of cycles per screen gratings can have so that Long Evans rats can reliably perform orientation discrimination. To relate our results to literature on visual acuity we tried to make an estimate of the decision distance in the touchscreen platform. The rats can discriminate between orientations with 70% accuracy up to 44 cycles per screen. This could roughly translates to the previously reported visual acuity of 1 c/degree assuming a viewing distance of 12.5 cm. This could be useful when designing new stimuli based on published results in c/degree. One could assume a viewing distance of 12.5 cm and expect similar discrimination performance in the touchscreen setup as in other tasks with a predefined viewing distance.

## 1. Introduction

Two-alternative forced choice tasks with freely moving animals have been used to study vision and cognition in rodents, from the initial experiments with a jumping stand [1] to the more recent visual water maze [2,3]. Both methods require the animal to make a distinction between two visual stimuli. To provide feedback to the animals, a correct choice is rewarded with food or by removal from the water, respectively. But these methods are labor intensive due to the frequent animal handling and limited number of trials that can be performed per day.

The touchscreen setup is an automated version of the visual water maze [4], that allows for testing of multiple animals in parallel with limited experimenter interaction. The animals are placed in an operant chamber for up to one hour and consecutive trials are started automatically or initiated by the animal. The touchscreen platform is commonly used to study executive functioning [5], learning and memory [6,7], and more recently vision [8,9]. The wide range of possible tasks, e.g., 5-choice serial reaction time task, discrimination and reversal learning [4], etc. allow for a high degree of flexibility in designing experiments. Further advantages include the limited experimenter interaction and appetitive reinforcement which reduce the animal’s stress levels [10]. Animals easily reach near 100% correct performance in these setups, and can learn very complicated visual tasks (e.g., [9]. The possibility of combining the behavioral task with electrophysiology or optogenetics further increases the benefit of the touchscreen platform for more in depth studies of cognitive and visual processes.

Unfortunately, there are also some downsides to studying vision with touchscreen setups. Typically relatively arbitrary and easy to discriminate black and white shapes are used. In visual neuroscience, however, more complex shapes with well-defined parameters are required. Typical stimulus parameters include spatial frequency (SF), orientation, contrast [11], velocity and direction of motion. Most of these can be easily defined on the screen, but when the animal is freely moving the position of the animals head cannot be controlled, which may distort the parameters at the moment of decision or response. The viewing distance and angle are for example important for determining the exact stimulus size, typically expressed in visual degrees. In vision experiments with freely moving animals there is usually a predefined decision boundary. This is not possible in the touchscreen platform since animals are allowed to freely explore, and only have to make their decision when they are right in front of the screen. It is thus not clear what the viewing distance is at the time the animal makes a decision.

The smallest feature that can be resolved by the visual system differs greatly between species and strains [12]. This visual acuity can be determined based on the sinusoidal grating with the highest SF that can still elicit a reliable discrimination, expressed as the number of cycles per degree (c/degree). The acuity for pigmented rats has been determined in different behavioral paradigms to be approximately 1 c/degree at optimal contrast [2,13,14], but is lower in albino rats [15]. These measurements have all been performed with horizontal or vertical gratings, for oblique gratings the acuity is only 0.7 c/degree [16], however this discrepancy disappeared after discrimination training with these oblique stimuli. In electrophysiological measurements in rat primary visual cortex the cut-off SF for a response is slightly higher at 1.2 c/degree [17,18].

Due to a growing interest in the use of a touchscreen platform in visual neuroscience, it is important to understand the limits of this system and of the tasks the animals can perform. To this end, we defined the visual acuity in rats while performing a pairwise discrimination task with gratings of increasing SF in the touchscreen platform. The SF of the stimuli is determined as it is presented on the screen and not as it is perceived by the animal, to overcome the absence of a clear decision boundary. Based on these measurements we can derive a theoretical decision distance assuming an acuity of 1 c/degree. Knowing the experimental limits of the platform can help in designing future touchscreen experiments focused on unraveling the visual system and its computations.

## 2. Materials and Methods

### 2.1. Stimuli

**Figure 1:**
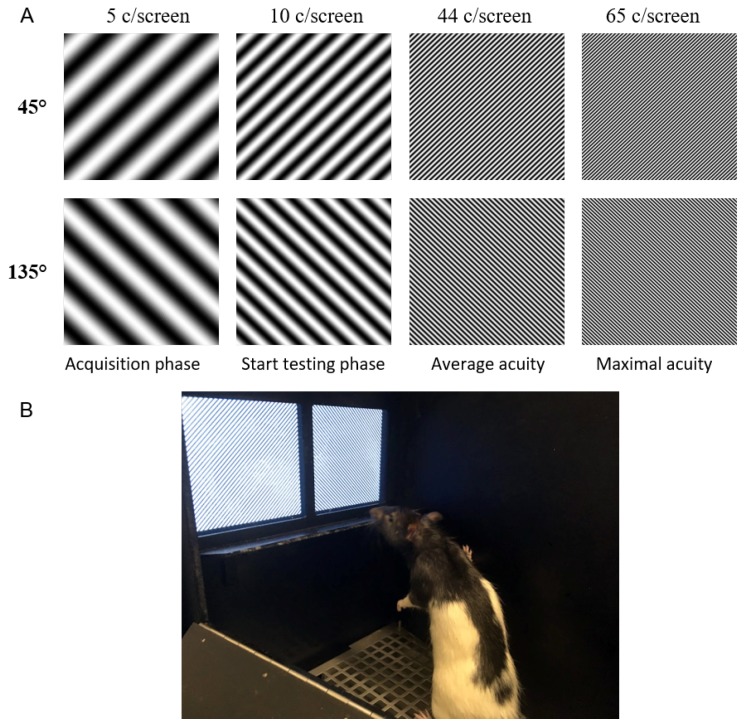
Example stimuli used in the testing phase. (**A**) Four example discrimination pairs shown to scale (30%). 5 and 10 c/screen are the lowest tested SFs, 44 c/screen is roughly the average acuity across animals (see results), and 65 c/screen is the highest acuity across animals. (**B**) Picture of rat in the touchscreen setup with 44 c/screen gratings.

Gray-scale sinusoidal gratings of 45° and 135° (diagonal gratings) with maximal contrast were used throughout the experiment (Figure 1). The luminance varied within a grating from 0.17 to 120 c/m^3^ but was not changed during the experiment. The spatial frequency, or size of the stimuli, is measured in cycles per screen (c/screen), and is an expression of the total number of cycles visible on the 9 × 9 cm screen at 0° orientation. For the acquisition phase a SF of 5 c/screen is used, or 2 cm for one full black and white cycle. During the testing phase the number of cycles is increased in steps of 5, 2.5 or 1.25 c/screen, decreasing the size of individual cycles.

### 2.2. Acquisition Phase: Habituation and Training

Eight male Long Evans rats were group housed (n = 4 per group) and trained in a pairwise discrimination task in the touchscreen system (Bussey-Saksida chamber, Camden Instruments). Animals were 14 weeks old at the start of experiments. All experiments were approved by the KU Leuven Ethics Committee (P119/2014).

Animals were trained in the touchscreen system according to a standardized protocol [6]. In short, animals were food deprived, receiving sucrose pellets during testing and standard food pellets after each session (10 g/day). Their body weight was monitored to remain above 85% of free feeding weight. The animals were first habituated to the setup and then followed the standardized shaping protocol through which they learned how to perform a simple discrimination task of a black versus white screen. Next, they were gradually trained in a pairwise discrimination task to discriminate 45° orversus 135° sinusoidal gratings of 5 c/screen. The rewarded orientation (S+) differed between the two cages (n = 4). A correct response (S+) resulted in a food reward and an incorrect response (S-) results in a 5 s time-out with the house light turned on. To facilitate learning and prevent side bias, incorrect trials were repeated until a correct response was obtained. These repetitions are not included in any analysis. Each trial was followed by a 20 s rest interval before animals were prompted to initiate the next trial. Once performance was above 85% on two consecutive days, the testing phase was started. Each session lasted for a maximum of 100 trials or 1 h, whichever came first.

### 2.3. Testing Phase: Increasing SF

To define the maximal SF that could be used for the pairwise discrimination tasks in the touchscreen platform, the SF was increased each session with 5 c/screen until performance dropped below a 70% threshold. Then the last SF with performance above 70% was retested and the SF was increased daily with 2.5 c/screen. This process was repeated once more with a step size of 1.25 cycles per screen, the last SF with a performance above 70% in this phase is considered the maximal possible SF. All averages are expressed as mean ± SEM. A gradual increase in task difficulty was used as this has been shown to improve learning as opposed to training with difficult stimuli [19].

### 2.4. Data Analysis

For each session the performance is determined as the percentage correct responses of all trials. A performance of 50% is expected by chance. A performance of at least 67% has to be obtained across 100 trials to show a deviation from chance with 80% power (one-sided binomial test). The criterion for discrimination was set slightly higher at 70% ( one-sided binomial test from 50%, *p* = $3.925\times 10^{-5}$; 95% CI = [0.62 1.00]).

Visual acuity was defined as the SF with an expected performance of 70%. This was determined by linear interpolation of the performance between the maximal SF and the last tested SF (performance <70%). This gives us the visual acuity in terms of cycles per screen.

Based on these results the visual acuity in cycles per degree (c/degree) could be estimated, by using the obtained acuity in c/screen and assuming several plausible viewing distances (5, 10, 15 and 20 cm) by following formula [20]):

(1) $$Acuity=VisualAngle^{-1}=\frac{1}{2*\tan^{-1}\frac{W}{2*D}}$$

where W refers to the width of one cycle (= screen size/cycles per screen), and D is the assumed viewing distance from the screen, both are expressed in cm. A correlation was performed between the learning rate and acuity of each animal. Welch’s *t*-test was performed to compare animals with a high and low acuity.

## 3. Results

### 3.1. Acquisition Phase

**Figure 2:**
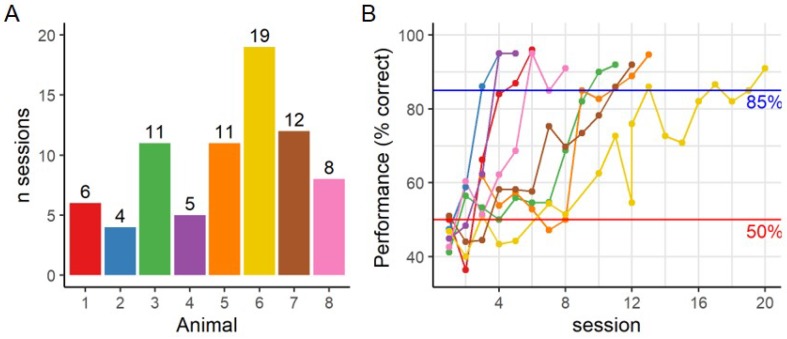
Acquisition phase. (**A**) Number of session required to reach criterion (>85% for 2 days) for each animal. (**B**) Individual learning curves with daily performance during this acquisition phase (5 c/screen). Colors refer to individual animals and remain consistent across figures. —85% criterion; —50% chance level.

After habituation and the standard shaping protocol in the touchscreen system, rats were trained to discriminate between two differently oriented gratings with 5 c/screen. On average rats needed 9.5 sessions to reach criterion (≥85%) (Figure 2), with a range of 4 to 19 training sessions. They reached an average performance of 90.6% ± 0.98 for the last two sessions. Figure 2B shows the different learning curves across animals.

### 3.2. Testing Phase

**Figure 3:**
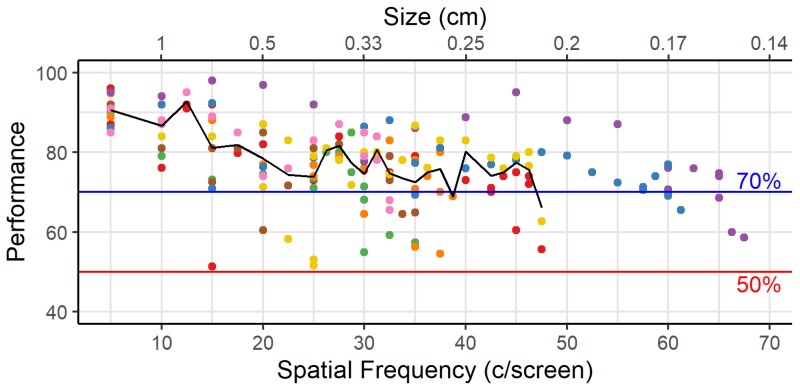
Testing phase. Average (–) and individual ( ∘ ) performance per session during the testing phase (starting from the last 2 days with 5 c/screen). Colors as in Figure 2A. After 50 c/screen the average is no longer indicated as only two animals are still included.

During the testing phase (Figure 3) there was a steady decrease in performance of all animals until 25 c/screen. At 30 c/screen some animals started to reach their acuity and are no longer tested on the higher SFs. From that point on the performance shown in Figure 3 was averaged across fewer animals.

**Figure 4:**
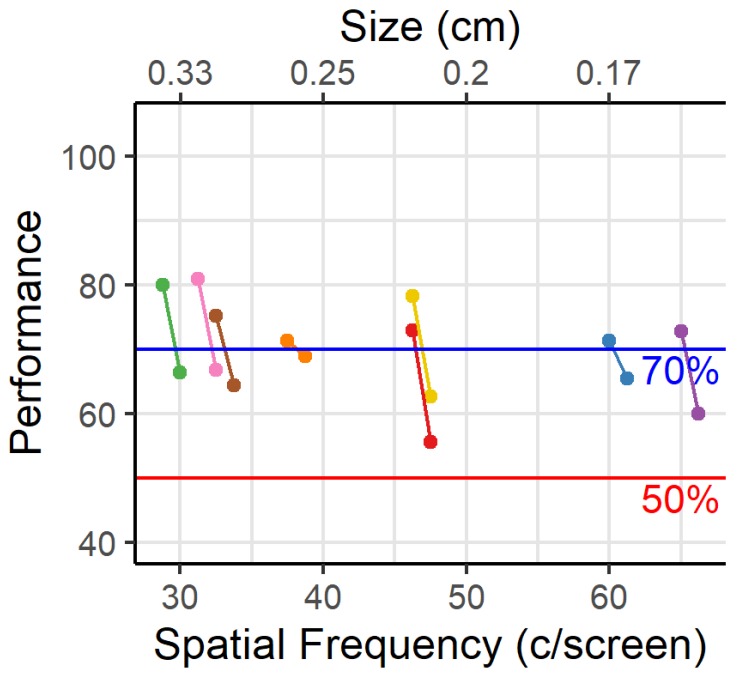
Visual acuity. Interpolation between the two SFs for which the performance was just above and below 70%. SF is shown per animal as c/screen (bottom axis) and as the width of one cycle (top axis).

Visual acuity can be defined by the maximal SF for which criterion was reached, or by interpolating the performance between this maximal SF and the next SF for which criterion was not reached (Figure 4). We performed interpolation for each animal between these last two SFs, that differed by 1.25 c/screen, to determine the SF likely to yield 70% performance. On average animals reach 70% performance at 44.02 c/screen, ranging from 29.67 to 67.28 c/screen.

To investigate the effect of visual acuity on the acquisition rate we performed a correlation analysis. There was no significant correlation (2-sided Pearson’s R = −0.459, t = −1.267, df = 6, *p*-value = 0.252) between acuity and the number of sessions needed for acquisition. When the outlier animal that needed 19 sessions (Figure 2A yellow, animal 6) in the acquisition phase is removed, there is a strong correlation (R = −0.857, t = −3.725, df = 5, *p*-value = 0.014), suggesting that learning might be impeded by the lower acuity of some animals.

Additionally, we looked at the difference in response speed between two groups of animals: those with a low acuity (animals 3 green, 5 orange, 7 brown and 8 pink) versus the ones with a higher acuity. Animals with a higher acuity took more time to respond to the stimuli (Welch’s two-sample t = 4.70, df = 187, *p*-value = $5.03*10^{-6}$) and needed longer to collect their reward (t = 3.732, df = 170, *p*-value = 0.0003). Thus, the animals with a high acuity respond in a less reflexive manner when the stimuli come on the screen.

**Figure 5:**
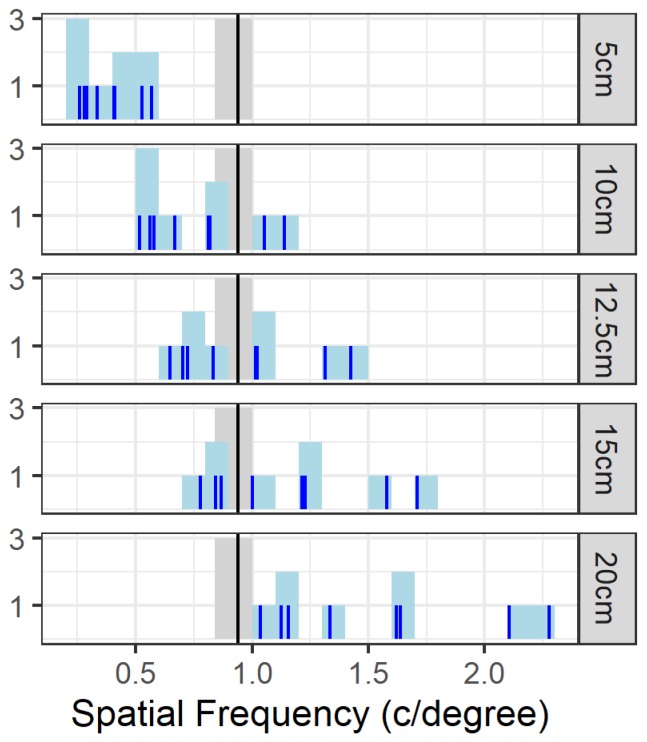
Visual acuity in c/degree. The SF with an expected performance of 70% (in c/degree) calculated based on several assumed viewing distances (in cm); shaded area: Acuity range with mean (|) according to [2].

To relate this data to previous studies in e.g., the visual water maze, we convert the visual acuity from cycles per screen to cycles per degree (c/degree), a more commonly used measure. To this end, we assume four viewing distances (5, 10, 15 and 20 cm) and calculated the cycles per degree based on the estimated acuity at 70% performance. A viewing distance of 10 cm would imply an average acuity of 0.77 c/degree (range = [0.52; 1.14] ), which is lower than earlier estimates in the visual water maze, which showed an average acuity of 0.94 c/degree (range = [0.84; 1.0] c/degree) [2]. With a viewing distance of 15 cm the acuity would be 1.15 c/degree (Figure 5) An intermediate viewing distance of 12.5 cm appeared to mimic the earlier data with an average of 0.96 c/degree and a range from 0.64 to 1.42 c/degree. It is important to not interpret this as a strict decision distance because it is possible that response strategies differ between animals. It is merely a proxy that would allow determination of stimulus features for future experiments based on published results in c/degree.

## 4. Discussion

Animals were trained in a pairwise discrimination task using the touchscreen setup. This method allows to test many animals at the same time while minimizing their stress-levels. Nonetheless, the platform does not allow to define a viewing distance, which is important in vision research. Typically, image dimensions are expressed in cycles per visual degree, to translate this to image dimensions on the screen one needs to know the exact viewing distance. We aimed at generating a bridge between these two techniques by defining the visual acuity, expressed as cycles per screen, in the touchscreen setup and relating this to previously published behavioral data on visual acuity in cycles per degree. This will allow us to compare the sensitivity of both methods and estimate an assumed viewing distance in the touchscreens.

**Figure 6:**
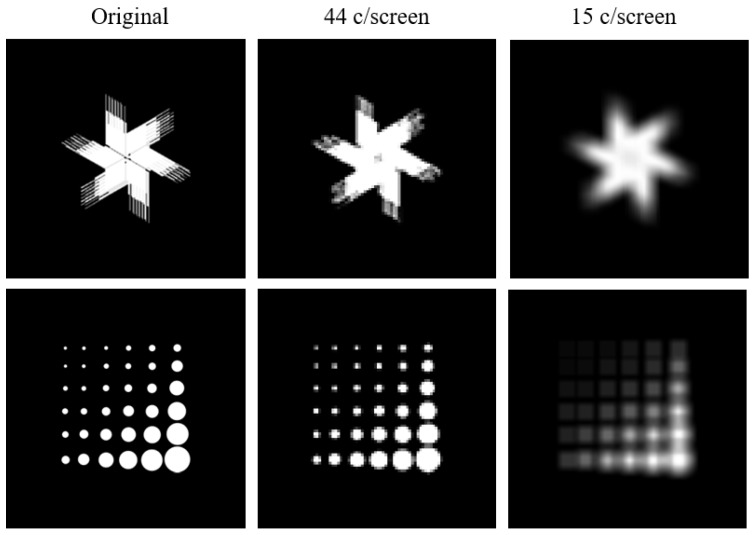
Commonly used training stimuli for the touchscreen setup, edited to reflect the limits of rat vision as defined by our acuity experiments. Pixel size is adjusted to reflect how the animals would perceive the images assuming a specific acuity.

The average visual acuity observed in rats is 44 c/screen. This means they can only resolve features that are larger than 0.1 cm, the associated width of half a cycle. To assess what effect this minimal dimension would have on the training stimuli that are included in the standard protocol of the touchscreen manufacturer, we adjusted the pixel size to 0.1 cm. When applying this assumption to the stimuli of the touchscreen platform, we see that smaller features are filtered out (Figure 6). Since both stimuli are sufficiently different, reliable discrimination is still possible. However, this does become important when designing new experiments and selecting stimuli for training. Stimuli should not contain any discrimination features that are smaller than the minimum size calculated here, to assure proper discrimination by the animal.

One important caveat to this exercise is that our threshold method determines the maximum spatial frequency that the animals can use to some degree, but it does not give us the spatial frequency that the animals typically use when multiple frequencies are present in images. Furthermore, this threshold is associated with a performance level of 70%, which is substantially lower than the 85% that we use as the criterion to conclude that an animal has mastered the task. Based upon Figure 3, if we want animals to obtain 85% accuracy on average, then we would rather work with the spatial frequency associated with approximately 15 c/screen.

Since the viewing distance can never be fully defined in the touchscreen platform, it is not advised to use measures based on viewing distance to define stimuli, e.g., cycles per degree. Therefore, we measured the cycles on the screen per stimulus. Based on these performances we could deduce that a decision distance of 12.5 cm for rats can be assumed when designing touchscreen experiments and relating to previously published data. This assumption only holds if there is no effect of experimental design on the visual acuity. It is possible that difference in motivation and task difficulty have influenced these measures of visual acuity. The assumption of viewing distance can thus only be applied to the touchscreen platform and should be reevaluated if used in other platforms. Even then these numbers should be used with caution.

To obtain a more universal estimate of the viewing distance for freely moving animals would require some adaptations to the setup and behavioral paradigm. It is for example possible to track the animal’s movements and to link stimulus presentation to the animal’s exact location. If the stimulus disappears when the animal comes too close to the screen, then it would force the animal to decide from a certain viewing distance. Alternatively, one could place a decision boundary within the setup and prevent the animal from approaching the screen up close. However, as our main interest was in the impact of stimulus features on task performance in the standard touchscreen setup and tasks, we decided to not deviate from the typical protocol and apparatus.

The maximal acuity in this experiment ranged from 32 to 67 c/screen. This range was much larger than expected from the literature. This could be due to the difference in incentive between the studies. Individual animals may respond differently to the food deprivation. Even though all animals undergo the same protocol, the amount of deprivation might still differ, and therefor also their intrinsic motivation to perform the task. Animals that are more severely deprived are expected to respond faster and collect their reward faster. In our data this is the case for the animals with the lowest acuity, indicating that a too strong deprivation might interfere with learning. Animals with a higher acuity took more time to engage in the task, which possibly enhanced learning. On the contrary, in the visual water maze all animals have the same incentive: getting out of the water to survive. Additionally, the experimental protocol for the touchscreens contains many more trials per daily session than in the visual water maze where only one trial is performed at a time. Animals need to perform multiple trials (n = 100) within an hour and we notice, in this and previous experiments, that response times and inter-trial intervals increase by the end of the session as animals become satiated. Finally, in the touchscreen setups the decision distance might differ between animals, while the decision distance is fixed in the water maze.

The estimated viewing distance based on these results is larger than expected when observing the animals’ behavior. Animals are able to approach the screen up to ∼1 cm before making the decision and indicating their response through a nose poke. Rats have been observed to spend several seconds in front of the screen before making a response. It is thus likely that the actual decision distance is only a few centimeters from the screen. The longer viewing distance could be a reflection of limitations of the set-up or the task difficulty.

Regarding task difficulty, typical acuity experiments use a detection task, where a sinusoidal grating needs to be distinguished from a gray screen [2,21]. In our task, we were not interested in the animal’s ability to detect a grating, but we were interested in the highest spatial frequency that could be used for a pairwise discrimination task in the touchscreen platform. In other words, we were interested in the smallest feature that could be used by the animals to solve the discrimination task. Animals may not pick up on the orientation differences or perceive the two stimuli at the same fine spatial frequency at which they can detect the presence of the gratings. This was shown in mice by the longer learning time for a discrimination task as compared to the detection task [21]. This may impede pairwise discrimination learning and testing in the later phases. Additionally, acuity for oblique stimuli is lower (0.7 c/degree) than for horizontal gratings in the visual water maze [16]. Although this probably reflects a lack of experience rather than a true difference in the animals perceptual abilities. After daily training with the oblique gratings for 14 days, the acuity was as high as for the horizontal gratings (Figure 2B from [16]). Orientation discrimination is a relatively difficult discrimination task. The estimated time for acquisition of a discrimination task is around 7 days when using the default stimuli from the training phases [6]. However, for our experiment the required sessions increased to more than 11 sessions for four out of eight animals. Figure 5 can be used to estimate the viewing distance if solving a discrimination task would make the animals rely upon lower spatial frequencies compared to a detection task. Suppose that this limit would be below or at 0.5 c/degree, then the viewing distance would decrease to less than 5 cm. This is consistent with how the animals approach the screens while solving the task.

In [2] the grating was presented at the end of one arm of the visual water maze. Making an error is punished by having to swim back to the correct arm and thus increasing the time in the water, which is a negative and stressful experience for the animals. Animals are encouraged to learn quickly and are discouraged from making a wrong decision. Even though only a few trials can be performed per day, this can give a quick read-out of visual acuity. On the contrary, our task is based on positive reinforcement, which improves animal welfare but may reduce their motivation and incentive. This reduced incentive in the touchscreen setup is seen in the slow learning curve of 7 days [6] as compared to the 2–4 session in the visual water task [2]. The same is observed in mice where it takes 8 days of 80 trials with a food reward [22] and only 20–40 trials for the visual water task [2]. Additionally, it should be noted that a lower performance is linked to a decreased amount of trials within one session (2-sided Pearson’s R = 0.83, *p* < 0.001 during the acquisition phase), as each trial with an incorrect response is repeated until a correct response is observed. These correction trials are not included in the trial count, and thus limit the amount of regular trials that can be performed within the hour. This same relationship holds during the testing phase (2-sided Pearson’s R = 0.18, *p* = 0.0179). However, with increased spatial frequency there is no change in the amount of trials (2-sided Pearson’s R = −0.08, *p* = 0.274) or in the number of correction trials needed to correct for each incorrect answer (2-sided Pearson’s R = 0.03, *p* = 0.725).

Both the reduced motivation and increased task difficulty may decrease our estimate of visual acuity threshold as compared to literature with other paradigms. However, this is a limitation inherent to the touchscreen setup, and should therefore not affect our results. As mentioned above, the conclusions about viewing distance and minimal feature size are unique to this setup.

In conclusion, the touchscreen platform can be used to study high-level vision when respecting its limitations. Stimulus presentation is not as well controlled as one may want, although we have shown that a small adjustment in experimental parameters may alleviate this problem. Despite this lack of control, we were able to obtain information about which amount of detail rats can use in touchscreen experiments. The resulting findings can help assess whether stimuli are appropriate for use in touchscreen experiments.
